# Biaxial Flexural Strength and Fracture Behavior of Five-Unit Monolithic Zirconia Fixed Dental Prostheses: An Experimental and Finite Element Study

**DOI:** 10.3390/jfb17100529

**Published:** 2026-10-19

**Authors:** Theresa Schwab, Jamila Yassine, Robert Nicic, Felix Borges, Alexander Pugachev, Franziska Schmidt

**Affiliations:** 1 Department of Prosthetic Dentistry, LMU University Hospital, LMU Medizin, Ludwig-Maximilians-Universität München, 80336 Munich, Germany; theresa.schwab@med.uni-muenchen.de; 2 Department of Prosthodontics, Geriatric Dentistry and Craniomandibular Disorders, Charité-Universitätsmedizin Berlin, Corporate Member of Freie Universität Berlin, Humboldt-Universität zu Berlin, 14197 Berlin, Germany; jamila.yassine@charite.de (J.Y.);; 3 SimQ GmbH, 85567 Grafing near Munich, Germany; fborges@simq.de (F.B.); apugachev@simq.de (A.P.)

**Keywords:** zirconia, fracture test, dental restoration, five-unit bridge, finite element analysis

## Abstract

Higher-translucency zirconia grades (4Y- and 5Y-PSZ) offer esthetic advantages over conventional 3Y-TZP but exhibit reduced flexural strength, raising concerns for restorations with reduced connector dimensions. It is unclear whether biaxial flexural strength, as determined by standardized specimen testing, translates to the fracture resistance of geometrically complex multi-unit bridges. This study compared the biaxial flexural strength (DIN EN ISO 6872:2015, *n* = 45) of three zirconia grades (3Y-TZP, 4Y-PSZ, and 5Y-PSZ) with the fracture behavior of monolithic five-unit fixed dental prostheses (FDPs) with deliberately undersized connectors (*n* = 24, teeth 33–37) made from the same three zirconia grades. Finite element (FE) analysis was used to compare the simulated elastic stress distribution with the experimentally observed fracture location. Biaxial flexural strength differed significantly among all groups (3Y-TZP: 1406.5 ± 133.3 MPa, 4Y-PSZ: 1146.9 ± 215.9 MPa, and 5Y-PSZ: 657.0 ± 137.5 MPa; *p* < 0.001). In contrast, bridge fracture loads differed less between the zirconia grades (3Y-TZP: 542.1 ± 44.9 N, 4Y-PSZ: 466.6 ± 116.6 N, and 5Y-PSZ: 372.1 ± 32.1 N). Only 5Y-PSZ showed significantly lower fracture loads than 3Y-TZP (*p* < 0.001), whereas 4Y-PSZ did not differ significantly from either 3Y-TZP (*p* = 0.253) or 5Y-PSZ (*p* = 0.129). All bridges initially fractured at the undersized connector, matching the site of the highest predicted tensile stress in den FE analysis. With this deliberately undersized connector, the differences in biaxial flexural strength between 3Y-TZP and 4Y-PSZ did not translate proportionally in a significant difference in fracture load. The FE analysis correctly identified the fracture-critical connector. The fracture load was not predicted, since the simulations were calibrated to the experimental fracture load values.

## 1. Introduction

Zirconia-based ceramics have emerged as a material for dental restorations due to their mechanical properties and biocompatibility [1,2]. Yttria-stabilized zirconia is available in various formulations, each with different mechanical and optical properties [3]. The translucency and mechanical strength of zirconia ceramics are inversely related, primarily due to their yttria content [4]. 3Y-TZP (3 mol% yttria-stabilized tetragonal zirconia polycrystal) demonstrates the highest flexural strength, ranging from 1000–to 1400 MPa. This strength primarily arises from stress-induced transformation toughening. In this process, crack propagation is impeded by the compressive stress generated when the material transforms from the tetragonal to the monoclinic phase. The addition of more than 3 mol% yttria results in the stabilization of cubic phase crystals alongside the tetragonal phase, creating partially stabilized zirconia (PSZ) materials. A decrease in the transformable tetragonal phase in these materials weakens transformation toughening, resulting in lower flexural strength. 4Y-PSZ, containing 4 mol% yttria, exhibits flexural strength values of 800–1000 MPa with an approximately 10% increased translucency compared to conventional 3Y-TZP. [3,5,6,7,8,9] Further increasing the yttria content to 5 mol% yields PSZ materials with flexural strength in the range of 600–800 MPa but achieving almost 25–30% higher translucency than 3Y-TZP [8,9,10,11].

These material differences influence clinical indications. Flexural strength is, besides chemical solubility, the main parameter for the classification as described in DIN EN ISO 6872:2015 [12]. According to this standard, high-strength 3Y-TZP with flexural strength values > 800 MPa belongs to Class 5. Therefore, it is suitable for monolithic prostheses and restorations with four and more units in all regions. Highly translucent 5Y-PSZ belongs to Class 4 ceramics and is recommended for maximum three-unit prostheses, in addition to more esthetic anterior applications. The higher translucency of PSZ also makes it more suitable for monolithic restorations without veneering [3,12,13,14].

In addition to the mechanical properties of zirconia, the structural design of restorations influences their load-bearing capacity [15,16,17]. For single crowns, the preparation design has been shown to considerably influence the fracture resistance [18]. In multi-unit fixed dental prosthesis (FDP), the connector regions represent critical areas, as their cross-sectional dimensions affect local stress concentrations. Previous studies have demonstrated that reducing the connector cross-sectional area increases stress and decreases the fracture load. Larger and more rounded connectors result in a more favorable stress distribution. These effects become more relevant with increasing prosthetic span, as longer-span restorations are subjected to greater bending moments [15,16,17,19]. Therefore, minimum connector cross-sectional area and limitations regarding the number of connected pontics are specified in the manufacturer’s recommendations for the respective zirconia grades [20]. In three-unit FDPs milled from a multi-layered zirconia blank, the positioning in the 4Y-PSZ or the 5Y-PSZ layer did not significantly affect fracture load. Most fractures originated in the connector region and propagated through the pontic [21].

Finite element (FE) simulations enable the prediction of stress distributions and the identification of the critical regions in dental restorations prior to fabrication [15,22]. In FDPs, such analyses consistently indicate that stresses concentrate at the connectors and that connector design influences the peak stress [15,23,24]. Moreover, finite element analyses of restorative materials have demonstrated that a restoration’s mechanical response depends on the interaction between the material and the restorative design. Differences in bulk material properties do not necessarily result in proportionally large differences in stress peaks at the restoration level [25].

To our knowledge, these studies did not compare several zirconia grades within one identical monolithic long-span geometry or compare the fracture locations to FE-predicted stress concentrations. The reliable application of such in silico methods in biomechanics, however, requires thorough verification and validation [26]. Recent FE literature emphasizes that the reliability and clinical interpretability of numerical findings depend strongly on modeling assumptions, including geometric representation, material stiffness, adhesive interface modeling, and boundary and loading conditions [27,28]. It is also emphasized that FE analysis should be considered part of a broader investigative strategy that integrates numerical modeling with laboratory experimentation [28]. Mechanical testing under realistic loading and boundary conditions, including fracture testing, is therefore necessary to provide such validation data [29]. Furthermore, establishing the correlation between simulated stress concentrations and experimentally observed fracture behavior is an essential step toward clinically applicable prediction tools [23,24].

The differences in strength between zirconia grades are well established, but it remains unclear whether strength rankings are still predictive when the same zirconia grades are used in a long-span geometry with a localized structural weak point. The aim of this study was to compare the biaxial flexural strength and the fracture load of 3Y-TZP, 4Y-PSZ and 5Y-PSZ in an identical monolithic five-unit FDP, to characterize their fracture patterns and to compare FE stress predictions with experimental fracture data. We hypothesized that (1) the ranking of zirconia grades (3Y-TZP > 4Y-PSZ > 5Y-PSZ) by biaxial flexural strength would not predict their ranking by fracture load in a monolithic five-unit FDP with a structural weak point, and (2) the location of maximum principal stress predicted by finite element analysis at the experimental fracture loads would correspond to the experimentally observed fracture-initiation site.

## 2. Materials and Methods

The study consisted of two experimental parts and a finite element simulation. First, the biaxial flexural strength of three zirconia grades was determined according to DIN EN ISO 6872. Additionally, fracture testing was performed on five-unit zirconia FDPs fabricated from the same zirconia grades. This setup was subsequently simulated in Ansys Mechanical. As the finite element analysis predicts elastic stress rather than actual fracture, an experimentally derived stress reference was required. Therefore, biaxial flexural strength testing with Weibull analysis was performed to evaluate the finite element predictions against the fracture data obtained from the FDPs.

Three different zirconia grades were investigated: Zolid ZI White (3Y-TZP), Zolid Gen-X (4Y-PSZ), and Zolid FX Multilayer (5Y-PSZ) (all from Amann Girrbach AG, Mäder, Austria). According to the manufacturer, Zolid ZI White contains 4.5–5.6 wt% Y_2_O_3_, has a three-point flexural strength of 1200 ± 150 MPa and is indicated for three-unit and multi-unit bridge frameworks. Zolid Gen-X contains 6.0–7.0 wt% Y_2_O_3_, has a reported three-point flexural strength of 1000 ± 150 MPa, and is indicated for crowns and for three-unit and multi-unit bridge frameworks. Zolid FX Multilayer contains 8.5–9.5 wt% Y_2_O_3_, has a reported three-point flexural strength of 700 ± 150 MPa, and is indicated for crowns and for bridge frameworks of up to three units extending into the molar region [30].

### 2.1. Biaxial Flexural Strength Test

Biaxial flexural strength testing was performed to establish a reference for evaluating the stress values obtained from the elastic finite element simulation. The biaxial flexural strength was determined using the piston-on-three-balls method in accordance with DIN EN ISO 6872:2015. A total of *n* = 45 specimens were tested, with 15 specimens in each group. The disk specimens were milled from pre-sintered zirconia blanks using a 5-axis CAM system (PrograMill PM7, Ivoclar Vivadent AG, Schaan, Liechtenstein) with PrograMill CAM software (version 4.4.13.0, Ivoclar Vivadent AG, Schaan, Liechtenstein). Following milling, sintering was performed in a dental furnace (Ceramill Therm 3, Amann Girrbach AG, Mäder, Austria), using the manufacturer’s standard program 1: heating from 200 °C to 1450 °C at 8 °C/min, holding at the maximum temperature for 2 h and then cooling to 200 °C over approximately 10 h. After sintering, the specimens were ground using coarse grinding paper (P500 WS Flex 18C, Hermes Schleifmittel GmbH, Hamburg, Germany), with a grain size of 30.2 ± 1.5 µm for 10 min minutes. This was followed by fine polishing using a 15 µm diamond suspension (Diamant-Suspension Magnum-Grün, Oberflächentechnologien Dieter Ziesmer, Trier, Germany) on a polishing cloth (ChemoMet polishing cloth, Buehler Ltd., Lake Bluff, IL, USA) for 10 min. The polishing process was carried out using a rotating polishing unit (Phoenix Alpha Grinder with Vector LC Power Head, Buehler Ltd., Lake Bluff, IL, USA).

After polishing, the disks had a mean diameter of 13.96 ± 0.02 mm and a mean thickness of 1.38 ± 0.01 mm. The thickness of each specimen was measured at three positions near the edge using a digital caliper (ABS, Fortis Einkaufsbüro Deutscher Eisenhändler GmbH, Wuppertal, Germany), and the mean value was used for the strength calculation. The diameter was measured at one position with the same digital caliper.

Each specimen was positioned on three 3 mm radius steel balls, arranged in a circle of 5.5 mm radius. A flat piston with a radius of 0.8 mm was used to apply load to the center of the disk until fracture occurred. To ensure even force distribution, 0.6 mm-thick polyethylene foil was placed between the piston and the specimen, as well as between the specimen and the steel balls. The mechanical tests were performed on a universal testing machine (ZwickRoell Z010 RetroLine, ZwickRoell GmbH & Co. KG, Ulm, Germany), at a crosshead speed of 1 mm/min in air at room temperature until fracture. The maximum load at fracture, *P* (N), was recorded for each specimen. The biaxial flexural strength *σ* (MPa) was calculated according to DIN EN ISO 6872:2015:

(1) $$\sigma =-0.2387\cdot P\cdot \frac{X-Y}{b^{2}}$$

with

(2) $$X=\left(1+\nu \right)\cdot \ln{\left(\frac{r_{2}}{r_{3}}\right)}^{2}+\left[\frac{1-\nu }{2}\right]\cdot {\left(\frac{r_{2}}{r_{3}}\right)}^{2}$$

(3) $$Y=\left(1+\nu \right)\cdot \left[1+\ln{\left(\frac{r_{1}}{r_{3}}\right)}^{2}\right]+\left(1-\nu \right)\cdot {\left(\frac{r_{1}}{r_{3}}\right)}^{2}$$

where *P* is the fracture load (N), *b* the specimen thickness at the fracture origin (mm), *ν* the Poisson’s ratio (0.25), $r_{1}\;$ the radius of the support circle (5.5 mm), $r_{2}$ the radius of the piston tip (0.8 mm), and $r_{3}$ the specimen radius (mm).

### 2.2. Fracture Test

The fracture test consisted of *n* = 24 five-unit bridges, with eight specimens fabricated for each of the zirconia grade. The FDP design was based on an STL file of a phantom model (Charité-Universitätsmedizin Berlin) and created using exocad DentalCAD (version FR-2024-09-27, exocad GmbH, Darmstadt, Germany). The abutment teeth 33 and 37 were prepared with shoulder (step) preparation. The five-unit fixed dental prosthesis spanned teeth 33 to 37 in the mandibular left quadrant from canine to second molar. The bridge consists of three pontics (teeth 34, 35, 36) between the two abutment crowns. A 7 mm diameter spherical indentation was incorporated into the occlusal surface of pontic 35 to improve the stability of the ball during strength testing.

According to the manufacturer’s recommendations, a minimum connector cross-sectional area of >12 mm^2^ is required for posterior FDPs with two connected pontics. To promote failure at a predefined location, the connector between teeth 34 and 35 was deliberately designed with a cross-sectional area below this recommended minimum. Thus, the fixed dental prostheses have an experimentally weakened geometry and are not clinically recommended. The connector geometries and dimensions are provided in Table 1 [20].

The bridges were milled and sintered from pre-sintered zirconia blanks using the same machines and settings as those used for the specimens of the biaxial flexural strength test. All objects were positioned at the central height of the blank and two blanks were used per zirconia grade. After sintering, all bridge surfaces were polished to a high-gloss finish using a two-stage protocol with diamond polishing instruments (EVE RA Universal polishers 0.2 μm, EVE Ernst Vetter GmbH, Pforzheim, Germany; Bison polishing brush with Zirkopol paste, Renfert GmbH, Hilzingen, Germany) at 10,000 rpm. The internal abutment surfaces were sandblasted with 25–70 μm aluminum oxide (Perlablast micro, Renfert GmbH, Hilzingen, Germany) at 1.5 bar for 10 s each internal abutment surface at a nozzle distance of 5 cm and an angle of 90° to ensure a stable cement bond.

Custom abutments serving as mechanical fixtures replicating teeth 33 and 37 were designed based on the bridge’s internal geometry with an appropriate cement gap and fabricated from glass-filled resin (Rigid 10K Resin, Formlabs Inc., Somerville, MA, USA) via stereolithography (Form 3B+, Formlabs Inc., Somerville, MA, USA). The abutments were integrated into a standardized carrier that ensured stable positioning of the specimens during testing. Thus, the test setup does not simulate the clinical supporting teeth but rather a standardized test fixture. The bridges were cemented onto the abutment carriers using resin-based dental cement (Panavia V5, Kuraray Noritake Dental Inc., Tokyo, Japan) following the manufacturer’s instructions. The assembled specimens were then embedded in cylindrical pots using cold-curing acrylic resin (Technovit 4071, Kulzer GmbH, Hanau, Germany; powder:liquid ratio 5:3) to ensure rigid fixation during mechanical testing.

Mechanical fracture testing was performed using a universal testing machine (ZwickRoell Z010 RetroLine, ZwickRoell GmbH & Co. KG, Ulm, Germany). Specimens were mounted via a central screw and alignment marks to ensure reproducible positioning. Load was applied through a 6 mm diameter steel sphere positioned in the spherical indentation on pontic 35 (Figure 1). Testing was conducted in two consecutive loading cycles at a crosshead speed of 1 mm/min, with a 1 N preload and automatic termination at 50% force drop. In the first cycle, specimens were loaded until initial crack formation, which was defined as the first force drop of 50% in the force-displacement curve. The specimens remained in the fixture, and loading was restarted with identical parameters. The second cycle was performed solely to propagate the crack to complete failure so that the fracture became fully visible. Only the first cycle was used for the determination of fracture load and displacement and for the comparison with the FE analysis results.

### 2.3. Finite Element Simulation

For the finite element analysis, experimental reproducibility was first verified by comparing 3D scans of two cemented bridges on their abutment carriers in CloudCompare (version 2.13.2). The signed deviation between the two scans was −0.017 ± 0.188 mm (RMS 0.19 mm), with the largest deviations located at the abutment carriers. The bridge geometry was then aligned to this validated experimental position and checked visually with a tolerance of ±0.2 mm. The complete test assembly, including the embedding material, test pot, and steel ball, was reconstructed in Ansys SpaceClaim 2024R1 (Ansys, Inc., Canonsburg, PA, USA; (Figure 2). The carrier and abutments were separated and simplified into two parts. Discretization was performed in ICEM CFD 2024R1 (Ansys, Inc., Canonsburg, PA, USA), with 4,307,565 elements at 1,541,933 nodes. The bridge was meshed using a hybrid approach combining tetrahedral elements with 7-layer prism inflation. The first prism layer had a height of 0.03 mm with a growth factor of 1.2. Surface element size in the connector region was approximately 0.2 mm. No formal mesh-convergence study was performed. Mean mesh quality was 0.79, with 98.6% of elements exceeding the 0.35 threshold. Abutments and CAD geometries (abutments, carrier, embedding, pot, ball) were meshed with 10-node tetrahedral elements, maintaining quality values > 0.35.

Isotropic elastic material models were applied to all components. Zirconia bridges were assigned an elastic modulus of 210 GPa and a Poisson’s ratio of 0.25. These values were used for all zirconia grades according to the manufacturer’s specifications. Since no failure criterion was implemented, the predicted stresses were compared with the biaxial flexural strengths for interpretation only. Abutments and carriers (Rigid 10K Resin, Formlabs Inc., Somerville, MA, USA) used E = 10 GPa and ν = 0.37. The embedding material (Technovit 4071, Kulzer GmbH, Hanau, Germany; powder:liquid ratio 5:3) was modeled with E = 2.3 GPa and ν = 0.35, while steel components (pot and ball) employed E = 200 GPa and ν = 0.30. Bonded contacts with Pure Penalty formulation were applied to all fixed component connections. The abutment-carrier interface employed Multi-Point Constraint formulation. Frictionless contact was defined between the ball and bridge. The cement layer (Panavia V5) used experimentally was not modeled as a separate body. Instead, the bridge was bonded directly to the abutments, assuming ideal adhesion. This is considered a modeling simplification. The test pot was fixed at its base and bore. Displacement-controlled loading was applied vertically to the ball surface, with magnitudes adjusted per zirconia grade to match experimental fracture loads. Static structural analysis was performed using Ansys Mechanical 2024R1 (Ansys, Inc., Canonsburg, PA, USA) with automatic substep subdivision and large deformation effects activated.

### 2.4. Statistical Analysis

Biaxial flexural strength was calculated according to DIN EN ISO 6872:2015 from the recorded fracture loads and specimen geometry. Normality of the data was assessed for each group using the Shapiro–Wilk test, and homogeneity of variances using Levene’s test (median-centered, Brown–Forsythe). As both assumptions were met, differences among zirconia grades were analyzed by one-way analysis of variance (ANOVA) with the zirconia grade as the fixed factor, followed by Tukey HSD post hoc comparisons (*α* = 0.05).

Weibull analysis was performed separately for each group (*n* = 15) according to DIN EN ISO 6872:2015 (Annex B). Specimens were ranked in ascending order of strength and assigned a failure probability with $P_{f}=\left(i-0.5\right)/N$. The data were linearized as $\ln\ln[1/(1\;-P_{f})]$ versus $\ln\left(\sigma \right)$ and fitted by linear regression. The Weibull modulus (*m*) was taken as the slope of the regression line. The characteristic Weibull strength (*σ_0_*) was obtained by setting *y* = 0 and calculating $\sigma_{0}=\;e^{x}$. All specimens were included in the analysis [12].

For the analysis of the fracture load data (*n* = 24), the normality was assessed using the Shapiro–Wilk test and homogeneity of variances using Levene’s test (median-centered, Brown–Forsythe). As variances were unequal, differences in fracture load between the groups were analyzed using Welch’s ANOVA followed by Games–Howell post hoc tests. The level of significance was set at *α* = 0.05 for all tests. All statistical analyses were performed using R software (version 4.5.1, R Foundation for Statistical Computing, Vienna, Austria).

## 3. Results

### 3.1. Biaxial Flexural Strength Test Results

All specimens (*n* = 15 each for 3Y-TZP, 4Y-PSZ, and 5Y-PSZ) fractured abruptly without visible plastic deformation. Fracture patterns were comparable across groups and characterized by fragmentary failure originating from the loading center with multiple wedge-shaped fragments. The mean biaxial flexural strength, the range of measured values, and the Weibull parameters of the three groups are summarized in Table 2. Flexural strength decreased with increasing yttria content (3Y-TZP: 1406.5 ± 133.3 MPa; 4Y-PSZ: 1146.9 ± 215.9 MPa, and 5Y-PSZ: 657.0 ± 137.5 MPa).

The Shapiro–Wilk test confirmed normal distribution of biaxial flexural strength values for all three groups, as no significant deviations from normality were detected (3Y-TZP *p* = 0.852, 4Y-PSZ *p* = 0.074, and 5Y-PSZ *p* = 0.479). Levene’s test indicated homogeneous variances (F(2, 42) = 1.71, *p* = 0.193). One-way analysis of variance revealed a statistically significant effect of zirconia grade on biaxial flexural strength (F(2, 42) = 78.28, *p* < 0.001). Post hoc Tukey HSD multiple comparisons demonstrated that all groups differed significantly from each other. The 4Y-PSZ group exhibited significantly lower flexural strength compared to 3Y-TZP (mean difference 259.6 MPa, 95% CI 111.8–407.4 MPa, *p* < 0.001). The 5Y-PSZ group showed significantly lower values than both 3Y-TZP (mean difference 749.6 MPa, 95% CI 601.7–897.4 MPa, *p* < 0.001) and 4Y-PSZ (mean difference 489.9 MPa, 95% CI 342.1–637.7 MPa, *p* < 0.001).

The characteristic Weibull strength decreased with increasing yttria content, and the Weibull modulus was highest for 3Y-TZP (*m* = 12.6) and lower for 4Y-PSZ (*m* = 6.2) and 5Y-PSZ (*m* = 4.9). Given the limited sample size (*n* = 15 per zirconia grade) and the presence of one low outlying value in 5Y-PSZ (315.8 MPa), which was retained in the analysis, the Weibull modulus should be interpreted with caution.

### 3.2. Fracture Test Results

#### 3.2.1. Fracture Load and Deformation

The mean fracture loads and the corresponding mean displacements at maximum load of the three groups are summarized in Table 3. Normal distribution was confirmed for all three zirconia grades (3Y-TZP *p* = 0.204, 4Y-PSZ *p* = 0.386, and 5Y-PSZ *p* = 0.721). However, Levene’s test indicated unequal variances between the groups (F(2, 21) = 8.95, *p* = 0.002). Welch’s ANOVA revealed a significant effect of zirconia grade on fracture load (F(2, 12.42) = 36.37, *p* < 0.001). Games–Howell post hoc tests showed that 5Y-PSZ differed significantly from 3Y-TZP (mean difference 170 N, 95% CI 118–222 N, *p* < 0.001). No significant differences were found between 3Y-TZP and 4Y-PSZ (mean difference 75.5 N, 95% CI -47.7–199 N, *p* = 0.253) or between 4Y-PSZ and 5Y-PSZ (mean difference 94.5 N, 95% CI -27.5–216 N, *p* = 0.129).

#### 3.2.2. Fracture Pattern

In the first loading cycle, specimens were loaded until initial crack formation. All specimens exhibited an initial crack on the gingival side of connector 34/35. This location corresponded to the deliberately weakened cross-section and the tension side of the connector, and it was reproducible across all zirconia grades. The fracture line was predominantly vertical through the connector, then horizontal in the occlusal direction along the indentation margins toward tooth 35.

In contrast to the consistent initiation site, the second loading cycle, complete failure of the FDP and the abutment revealed differences between the zirconia grades. In the 3Y-TZP bridges, the secondary failure occurred at the resin abutment 37, which fractured horizontally in seven cases (Figure 3a). In specimen 3Y-B5 the connector 36/37 failed instead. The 4Y-PSZ group showed heterogeneous failure patterns. Connector 34/35 failed consistently, but the secondary fracture sites varied between abutment 37 (4Y-B1) and connector 36/37 (4Y-B2, 4Y-B3), five specimens showing only localized chipping or an isolated connector fracture (Figure 3b). All 5Y-PSZ bridges exhibited uniform failure patterns with fractures occurring at both connectors 34/35 and 36/37, while abutment 37 remained intact in all cases (Figure 3c).

### 3.3. Finite Element Simulation Results

Displacements were adjusted to reproduce the mean experimental fracture loads of each group. Vertical displacements of 0.072 mm, 0.064 mm, and 0.055 mm were applied to the load sphere for 3Y-TZP, 4Y-PSZ, and 5Y-PSZ, respectively. These prescribed displacements resulted in reaction forces of 546 N for 3Y-TZP, 467 N for 4Y-PSZ, and 375 N for 5Y-PSZ, showing close agreement with the experimental mean values of 542 N, 467 N, and 372 N, with deviations below 5 N in all cases (Table 4).

Stress analysis revealed highly localized concentrations at connector regions, while the remaining bridge structure exhibited minimal stress. Crown regions and outer connectors 33/34 and 36/37 exhibited stress levels below 50 MPa. Maximum principal stresses and von Mises equivalent stresses both peaked at connector 34/35, corresponding to the deliberately weakened cross-section where experimental fracture initiation occurred.

As zirconia is a brittle material, maximum principal stress was used as the primary evaluation criterion. Von Mises stress is reported as a complementary result. For the 3Y-TZP, maximum principal stress reached 1427 MPa on the inferior surface of connector 34/35, concentrated along the bucco-lingual axis (Figure 4a). Von Mises equivalent stress peaked at 1623 MPa on the superior surface of connector 34/35 toward the buccal aspect (Figure 4b). The adjacent connector 35/36 showed a secondary stress concentration of approximately 650 MPa (von Mises equivalent stress) on the inferior surface. Cross-sectional analysis demonstrated that critical stress extended beyond surface layers, with von Mises stress decreasing from 1623 MPa to approximately 300 MPa over a depth of 0.2 mm (Figure 4c).

Principal stress analysis revealed predominantly tensile stress on connector inferior surfaces and compressive stress on superior surfaces (see the signed equivalent stress plot in Figure 4d). Stress distribution from the loading site on pontic 35 followed the occlusal surfaces toward adjacent connectors, with particularly concentrated transmission through connectors 34/35 and 35/36.

Zirconia grade-specific analysis demonstrated similar stress distribution patterns but varying stress magnitudes corresponding to fracture load differences. The 4Y-PSZ simulation at 467 N showed a maximum principal stress of 1222 MPa and a von Mises stress of 1383 MPa. The 5Y-PSZ group at 375 N exhibited a reduced maximum principal stress of 985 MPa and a von Mises stress of 1108 MPa (Table 4).

## 4. Discussion

This study investigated the fracture load of three zirconia grades in a five-unit monolithic fixed dental prosthesis design and compared FE stress predictions with experimental fracture testing. The key findings demonstrate zirconia grade-dependent fracture loads (3Y-TZP: 542.1 ± 44.9 N, 4Y-PSZ: 466.6 ± 116.6 N, 5Y-PSZ: 372.1 ± 32.1 N). agreement of the predicted maximum stress location in the FE analysis corresponds with the fracture-initiation site in the experimental fracture load tests, which was at the deliberately undersized connector 34/35. 4Y-PSZ showed the greatest variability in fracture load, as reflected by the largest standard deviation, and also the most heterogeneous secondary fracture patterns, with failure occurring either at abutment 37 or at connector 36/37. Notably, the ranking of the bridge fracture loads only partially corresponded to the ranking obtained from the biaxial flexural strength measurements. Although the biaxial flexural strength differed significantly among all three zirconia grades, following the order 3Y-TZP > 4Y-PSZ > 5Y-PSZ (Table 2), fracture load differed significantly only between 3Y-TZP and 5Y-PSZ, whereas 4Y-PSZ did not differ significantly from either 3Y-TZP or 5Y-PSZ. This was the case despite a difference in mean biaxial flexural strength of almost 260 MPa between 3Y-TZP and 4Y-PSZ and almost 490 MPa between 4Y-PSZ and 5Y-PSZ. Possibly, the intentionally undersized 34/35 connector created a fracture-critical region, reducing the extent to which differences in bulk strength between 3Y-TZPand 4Y-PSZ and 4Y-PSZ and 5Y-PSZ were reflected in the fracture load of the FDPs. Since the dimensions of the connector were not varied in the present study, the contributions of the zirconia grade and geometry cannot be tested. This interpretation is, however, consistent with previous finite element studies in which connector dimensions and span length were identified as major determinants of fracture resistance in multi-unit zirconia restorations [15,23].

The fracture loads recorded in the present investigation were considerably lower than those previously reported for shorter-span restorations, where values above 1600 N have been described for three-unit 3Y-TZP bridges [17,19]. This difference likely reflects the combination of the extended five-unit span with three pontics and the deliberately reduced connector dimensions. The connector between units 34 and 35 had a cross-sectional area of 7.5 mm^2^ and was intentionally designed below the manufacturer’s recommended minimum to establish a reproducible fracture site. Therefore, the present results cannot be directly extrapolated to clinically recommended FDP designs. For five-unit restorations intended for clinical application, considerably larger connector cross-sections according to the manufacturer’s recommendation would be necessary [20].

The FE analyses showed good agreement with the experimentally observed fracture-initiation location. In all three simulations, the maximum stress concentration was located at connector 34/35, where crack initiation was observed in all specimens. The reaction forces were not predicted independently, because the prescribed displacements were adjusted to reproduce the mean experimental fracture loads with a difference of less than 5 N. 

Nevertheless, the simulated maximum principal stresses at failure ranged from 985 to 1427 MPa and were consistently higher than the corresponding mean biaxial flexural strengths measured for the same zirconia grade, which ranged from 657 to 1407 MPa (Table 2). The maximum principal stress was 1.4% for 3Y-TZP, 6.5% for 4Y-PSZ, and 50% for 5Y-PSZ higher than the mean biaxial flexural strength. Comparing the simulated maximum principal stress with the Weibull characteristic strength, the simulated values for 3Y-TZP and 4Y-PSZ were slightly lower. For 5Y-PSZ the simulated maximum principal stress was 35% higher as the Weibull characteristic strength. In a biaxial flexural strength test, a comparatively large specimen volume is exposed to relatively uniform tensile stresses. By contrast, the connector region of the bridge is characterized by a highly localized stress concentration within a much smaller critical volume. Because the probability of fracture increases with the volume exposed to stress close to the maximum value, a small and locally stressed region may tolerate a higher peak stress before failure than a larger bulk specimen [11]. This may provide an explanation for the simulated local stresses exceeding the experimentally determined bulk strength values reported in Table 2.

The analysis of the fracture patterns described in Section 3.2.2 also revealed group-dependent differences extending beyond the initial fracture site. The 3Y-TZP bridges displayed heterogeneous secondary fracture patterns, whereas 3Y-TZP and 5Y-PSZ bridges showed a more uniform failure pattern. Seven out of eight 3Y-TZP FDPs failed at abutment 37 and the 4Y-PSZ FDPs fractured at connectors 34/35 and 36/37. This may be related to the reduced transformation-toughening capacity reported in the literature for high-yttria PSZ. As the cubic phase content increases, the tetragonal-to-monoclinic phase transformation responsible for crack-tip shielding in 3Y-TZP becomes progressively restricted [8,9]. The reduced ability for crack arrest in 5Y-PSZ may therefore be an explanation for the more consistent fracture pattern observed in this group, however, this is an interpretation based on literature.

The agreement between the location of maximum principal stress and the fracture-initiation site supports the use of the elastic FE approach for comparative material evaluation and for identifying fracture-critical connector regions. This is in accordance with recommendations emphasizing the need for experimental verification and validation of in silico biomechanical models before their results are extrapolated to clinical conditions [26,29].

However, the vertical displacements required to reach the fracture loads were substantially lower in the FE simulations than in the experimental tests. The maximum simulated displacement was 0.072 mm, whereas the experimentally measured mean displacement values ranged from 0.43 to 0.49 mm. This difference is most likely related to the purely elastic material behavior assumed in the numerical model. The FE analysis did not account for crack initiation, microplastic deformation, or additional compliance arising from the cement layer and the abutment assembly used in the experimental setup. Consequently, the model appears suitable for identifying fracture-critical locations, but it cannot reproduce the complete load–displacement response preceding failure.

### Limitations

The findings of this study are subject to several limitations. The experimental investigation was limited to a single bridge geometry and span configuration tested under monotonic loading. The connector geometry was deliberately undersized to represent a mechanically weakened design and therefore should not be generalized as clinically recommended FDP designs. The influence of different connector geometries was also not assessed. Furthermore, the FDP groups had a relatively small sample size, which limited the statistical power. Cyclic fatigue, thermocycling, and exposure to a moist environment comparable to oral conditions were not included, although these factors may influence the long-term clinical performance of zirconia restorations. In addition, the abutments were represented by rigid resin specimens rather than natural teeth with periodontal support, which influences the distribution of stress.

The FE model was based on purely linear-elastic material properties and did not explicitly simulate fracture. Identical elastic properties were assumed for all zirconia grades. No formal mesh-convergence study was performed. However, the location of maximum stress and relative comparison between different zirconia grades are not affected by this limitation. The cement layer (Panavia V5) was not modeled as a separate component, as the bridge was bonded directly to the abutments. This idealization neglects the compliance of the cement layer and assumes perfect adhesion. Consequently, the numerical model is likely stiffer than the experimental setup, which probably contributes to the observed discrepancy in displacement. In addition, the absence of the cement layer may alter local stress distributions at the bridge-abutment interface. The biaxial strength values used as a reference were derived from standardized specimens under a comparatively uniform stress state, whereas the FE model represents a full bridge geometry with highly localized stress concentrations. Additionally, the findings for 5Y-PSZ should not be interpreted as clinical evidence, as this zirconia grade is not indicated for the present five-unit FDP design.

## 5. Conclusions

Within the limitations of this in vitro study and the deliberately weakened connector geometry, the difference in biaxial flexural strength between 3Y-TZP and 4Y-PSZ was not reflected in a statistically significant difference in FDP fracture load. In contrast, 5Y-PSZ exhibited both the lowest biaxial flexural strength and the lowest fracture load.

The elastic FE analysis, calibrated to the experimental fracture loads, correctly identified the fracture-critical connector regions and may serve as an assessment tool for stress localization in connector design. Because the model relies on linear-elastic behavior without a failure criterion, it does not predict fracture load and did not reproduce the complete experimental load–displacement behavior. Overall, the results are consistent with the importance of adequate connector dimensioning in extended-span zirconia restorations, particularly when highly translucent zirconia grades with lower mechanical strength are selected.

## Figures and Tables

**Figure 1:**
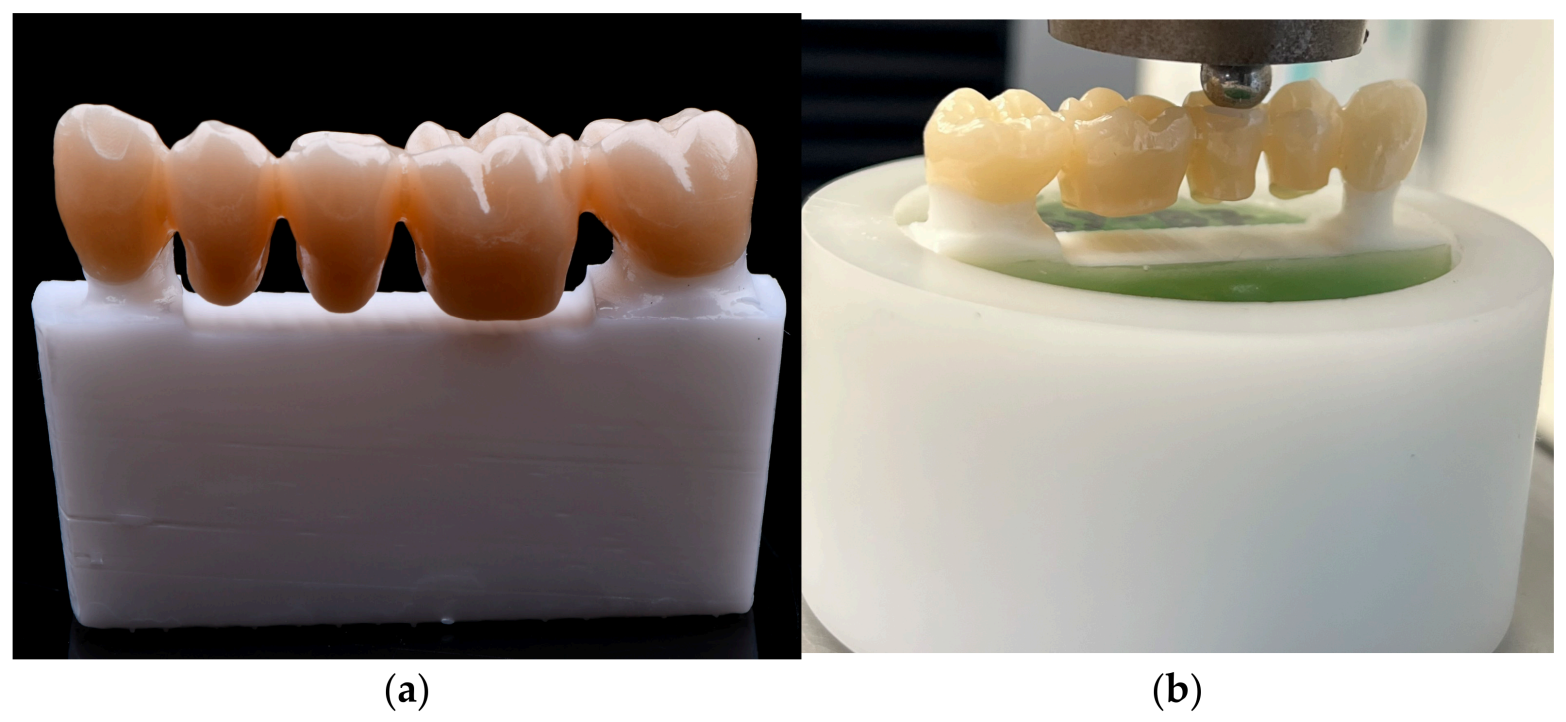
(**a**) Sintered and polished five-unit bridge cemented on a custom 3D-printed abutment carrier; (**b**) mechanical fracture testing with a 6 mm diameter steel sphere positioned at tooth 35.

**Figure 2:**
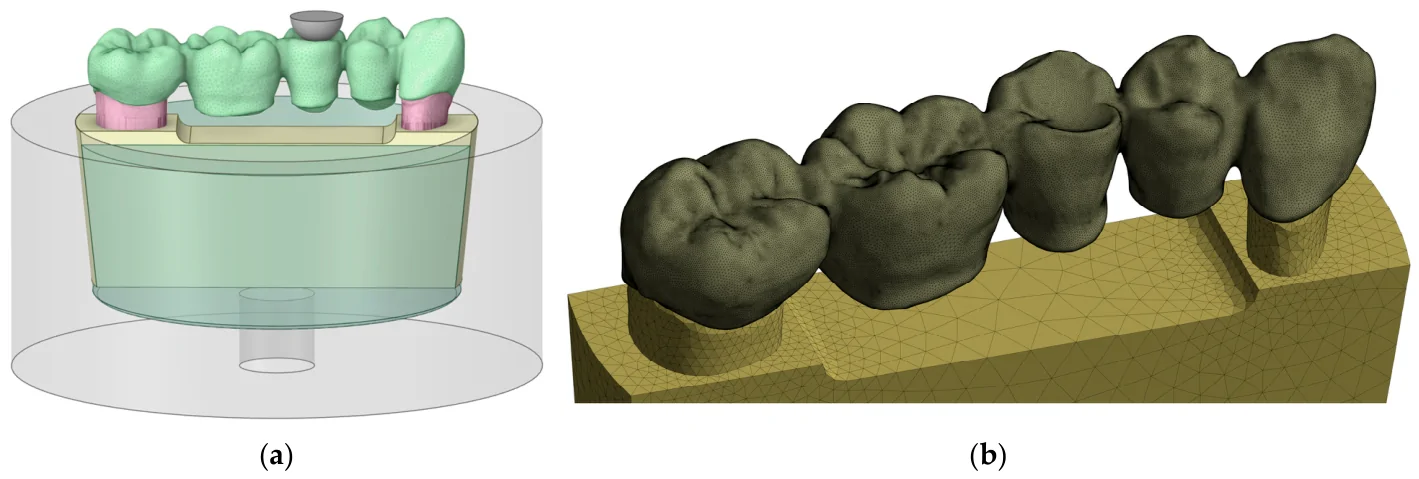
(**a**) Complete assembly of the Ansys setup with the test pot, embedding material, abutment carrier, abutments, bridge and steel ball; (**b**) meshing of the bridge and abutment.

**Figure 3:**
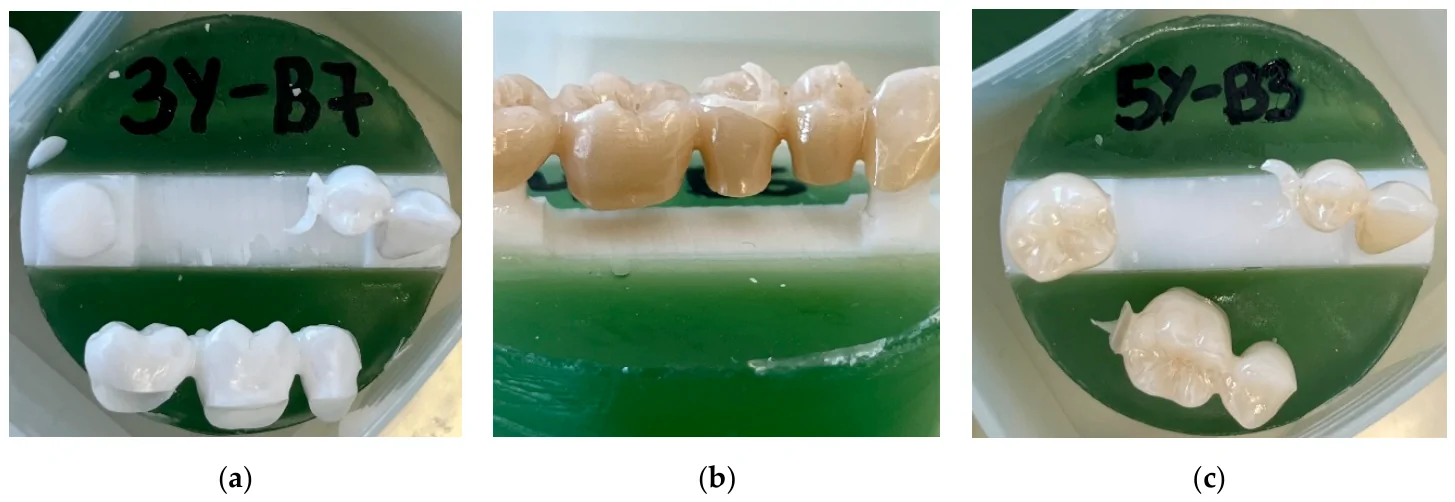
(**a**) Characteristic fracture pattern of the 3Y-TZP group using the example of bridge 3Y-B7; (**b**) possible fracture pattern of the 4Y-PSZ group using the example of bridge 4Y-B5; (**c**) characteristic fracture pattern of the 5Y-PSZ group using the example of bridge 5Y-B3.

**Figure 4:**
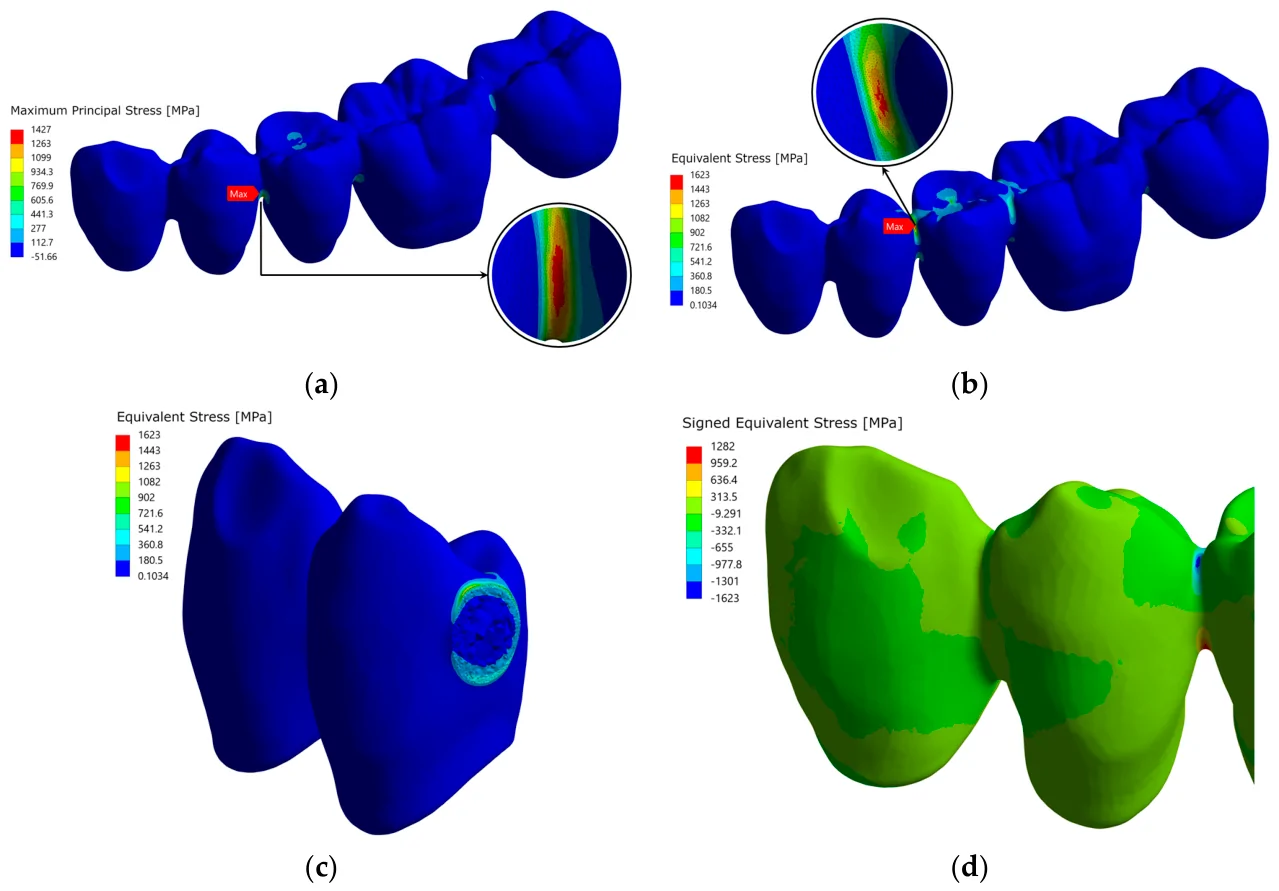
(**a**) Maximum principal stress distribution at fracture load for 3Y-TZP at 546 N; (**b**) von Mises equivalent stress distribution in 3Y-TZP bridge at 546 N; (**c**) cross-sectional stress gradient in connector 34/35; (**d**) principal stress distribution showing tensile stress on inferior surfaces and compressive stress on superior surfaces.

**Table 1:** Connector dimensions of the five-unit fixed dental prosthesis with an experimentally weakened geometry at connector 34/35.

Connector Location	Shape	Dimensions	Cross-Sectional Area
33/34	oval	5.1 mm height × 3.0 mm width	12.0 mm^2^
34/35	circular	3.1 mm diameter	7.5 mm^2^
35/36	circular	4.4 mm diameter	15.2 mm^2^
36/37	circular	4.4 mm diameter	15.2 mm^2^

**Table 2:** Biaxial flexural strength and Weibull statistics.

Zirconia Grade	Mean ± SD, MPa	Range, MPa	Weibull Characteristic Strength, MPa	Weibull Modulus
3Y-TZP	1406.5 ± 133.3	1184.4–1644.8	1460.3	12.6
4Y-PSZ	1146.9 ± 215.9	860.7–1535.0	1232.0	6.2
5Y-PSZ	657.0 ± 137.5	315.8–861.4	718.7	4.9

**Table 3:** Fracture loads and displacements at maximum load of the five-unit monolithic bridges.

Zirconia Grade	Fracture Load Mean ± SD, N	Fracture Load Range, N	Displacement Mean ± SD, mm	Displacement Range, mm
3Y-TZP	542.1 ± 44.9	483.4–594.0	0.49 ± 0.11	0.37–0.68
4Y-PSZ	466.6 ± 116.6	327.2–631.9	0.43 ± 0.10	0.33–0.57
5Y-PSZ	372.1 ± 32.1	334.0–430.5	0.45 ± 0.13	0.27–0.64

**Table 4:** Summary of finite element simulation results for the three zirconia grades.

Zirconia Grade	Displacement, mm	Reaction Force, N	Max. Principal Stress, MPa	Von Mises Stress, MPa
3Y-TZP	0.072	546	1427	1623
4Y-PSZ	0.064	467	1222	1383
5Y-PSZ	0.055	375	985	1108

## Data Availability

The original contributions presented in the study are included in the article, further inquiries can be directed to the corresponding author.
